# Long term impact of *PositiveLinks*: Clinic-deployed mobile technology to improve engagement with HIV care

**DOI:** 10.1371/journal.pone.0226870

**Published:** 2020-01-06

**Authors:** Chelsea E. Canan, Marika E. Waselewski, Ava Lena D. Waldman, George Reynolds, Tabor E. Flickinger, Wendy F. Cohn, Karen Ingersoll, Rebecca Dillingham

**Affiliations:** 1 Department of Medicine, University of Virginia School of Medicine, Charlottesville, VA, United States of America; 2 Health Decision Technologies, Oakland, CA, United States of America; 3 Department of Public Health Sciences, University of Virginia School of Medicine, Charlottesville, VA, United States of America; 4 Department of Psychiatry and Neurobehavioral Sciences, University of Virginia School of Medicine, Charlottesville, VA, United States of America; Johns Hopkins School of Public Health, UNITED STATES

## Abstract

**Background:**

PositiveLinks (PL) is a smartphone-based platform designed in partnership with people living with HIV (PLWH) to improve engagement in care. PL provides daily medication reminders, check-ins about mood and stress, educational resources, a community message board, and an ability to message providers. The objective of this study was to evaluate the impact of up to 24 months of PL use on HIV viral suppression and engagement in care and to examine whether greater PL use was associated with improved outcomes.

**Setting:**

This study occurred between September 2013 and March 2017 at a university-based Ryan White HIV clinic.

**Methods:**

We assessed engagement in care and viral suppression from study baseline to the 6-, 12-, 18- and 24-month follow-up time periods and compared trends among high vs. low PL users. We compared time to viral suppression, proportion of days virally suppressed, and time to engagement in care in patients with high vs. low PL use.

**Results:**

127 patients enrolled in PL. Engagement in care and viral suppression improved significantly after 6 months of PL use and remained significantly improved after 24 months. Patients with high PL use were 2.09 (95% CI 0.64–6.88) times more likely to achieve viral suppression and 1.52 (95% CI 0.89–2.57) times more likely to become engaged in care compared to those with low PL use.

**Conclusion:**

Mobile technology, such as PL, can improve engagement in care and clinical outcomes for PLWH. This study demonstrates long-term acceptability of PL over two years and provides evidence for long-term improvement in engagement in care and viral suppression associated with PL use.

## Introduction

Over 60% of new HIV infections in the United States are transmitted by people who are aware that they are living with HIV but who are not engaged in care or who have not achieved HIV viral suppression [1]. Proper adherence to effective antiretroviral treatment allows people living with HIV (PLWH) to maintain a suppressed viral load, leading to improved health outcomes for the individual and preventing further HIV transmission to others. Standard-of-care calls for routine HIV appointments, approximately every six months, and consistent adherence to daily oral medications. Despite the availability of effective treatment, many PLWH continue to live with an unsuppressed HIV viral load due to lack of engagement in care or improper adherence to prescribed treatment regimens. Effective, durable, and scalable strategies for helping PLWH overcome personal and structural barriers to engaging with HIV care are urgently needed.

Mobile technology (mHealth) offers unique opportunities to support chronic disease management, including the care of PLWH. mHealth may be an especially important tool to address disparities in chronic disease outcomes due to high acceptability and accessibility of mobile-based technologies for underserved populations [2,3], including those who are disproportionately affected by the HIV epidemic and are less likely to achieve key clinical outcomes like sustained viral suppression [4]. Through features such as appointment reminders and accessible communication channels with providers, mHealth has been shown to help engage patients lost to or at risk of being lost to care by providing information more efficiently and effectively [5–11]. Further, features such as medication reminders and self-monitoring of behaviors have been associated with improvements in engagement in care and clinical outcomes [8,12, 13–20,21–23].

PositiveLinks (PL) is a clinic-based mobile platform designed to increase engagement in care among PLWH. Details of the development of PL have been published elsewhere [24]. Briefly, PL was developed using evidence-based principles for chronic disease self-management and iterative formative research with the target clinic population. PL has a patient mobile app that contains features such as medication reminders, mood and stress check-ins, provider messaging, informational resources, and an anonymous community board that allows users to interact with one another [24]. Results from a prospective observational pilot study demonstrated an improvement in viral suppression and engagement in care among PL users 12 months after receiving the app [25]. Subsequently, a revised version of PL was developed and deployed with the additional features and functionality suggested by original patient users, and more patients were enrolled into an additional study of the platform’s use and potential impacts.

This current study was designed to provide further evidence for the impact of PL on clinical outcomes over a longer timeframe. In this paper, we evaluate the impact of PL on engagement in care and HIV viral suppression for up to two years. In addition, to test the hypothesis that greater engagement with PL is associated with greater improvement in clinical outcomes, we compare outcomes among high and low users of PL.

## Methods

### Setting

This study took place at a university-based Ryan White HIV Clinic between 2013 and 2017. The study site serves HIV patients from a wide geographic area, including both suburban and rural regions, many of whom face structural and personal barriers to care typical of nonurban patient populations including transportation, stigma, and financial barriers. HIV care providers at the clinic recruited patients into the PL study who they assessed to be at high risk for loss to care and, as such, were more likely to be non-adherent to medication and follow-up instructions, or patients who were newly initiating or restarting care at the clinic. Patients at the clinic who are initiating or returning to care are offered a medical case management assessment at the first medical appointment. The medical case manager evaluates potential barriers to engagement in care and provides referrals or services when possible, such as transportation or food assistance. If barriers are identified that require additional assistance, patients are also offered the option of working with a clinic-based community health worker who can work in the community with patients to overcome barriers. Eligibility criteria included being at least 18 years of age, diagnosis of HIV, receiving care at the Ryan White HIV clinic, and willing to participate in the PL program. This study was reviewed and approved by the university’s Institutional Review Board, and all patients provided written informed consent to participate.

### PositiveLinks intervention

Two iterations of the PL app were used during this study: PL 1.0 (launched September 2013) and PL 2.0 (launched June 2016). Details of the development of PL 1.0 and its preliminary findings have been previously published, including a summary of early formative and pilot work and qualitative assessments of PL users and features [24–28]. PL 2.0 was developed as a result of feedback from usability interviews with PL participants (“members”) and providers. Both PL versions contain educational resources about HIV, health and wellness, and social determinants of health, a community board where members can interact anonymously with one another, and daily ecological momentary assessment (EMA) queries that ask members to rate their current mood (1: very sad to 5: very happy), stress level (1: very low to 5: very high), and self-reported medication adherence (yes or no). These three EMA queries encourage consistent PL use by prompting members to access the app daily. The primary modification to PL 2.0 was the addition of a secure messaging feature that allows PL members to communicate directly with their HIV clinic providers for clinical or appointment questions and members of the PL team for technical or program-related questions.

Members who enrolled in PL 1.0 continued to use the app until they chose to discontinue or until the launch of PL 2.0, at which point PL 1.0 was terminated and members were offered the option to enroll in PL 2.0. We measured PL utilization monthly following each member’s initial enrollment in PL for all months that the member was enrolled. All members in PL 1.0 received a monthly phone credit for their cell phone bill. Beginning with PL 2.0, members only received the monthly phone credit if they responded to 48% or more of the daily EMA queries the previous month. This level of participation was established in consultation with the project funder as a marker of sufficient engagement with the program to merit continued phone credit support and is consistent with other expectations relative to visit attendance, for example, for receipt of transportation support.

### Outcomes

Primary outcomes for this study were engagement in care and HIV viral suppression. Engagement in care was assessed at baseline and at 6-month intervals following the patient’s first enrollment in PL. Engagement in care was defined using the contemporaneous Health Resources & Services Administration (HRSA) metric: attendance at 2 or more appointments separated by at least 90 days within the past year. HIV viral load data was collected as clinically indicated, at approximately 6-month intervals. Viral suppression was defined as having a HIV viral load <200 copies/mL. Members were included in the clinical outcomes analyses for all time periods in which they were still enrolled, regardless of their level of engagement in PL. Usage statistics are reported only for members who were actively using the app.

### Statistical methods

We tested for significant differences in baseline characteristics between PL members who had a 48% or greater response rate to daily queries within the first month of PL enrollment compared to those with a response rate less than 48% using chi-square or Fisher’s exact tests for categorical variables or t-tests for continuous variables. Characteristics included age, sex, race/ethnicity, insurance status, income, level of education, months from HIV diagnosis to PL enrollment, perceived stress, HIV-related stigma (Berger HIV Stigma scale [29]), social support (Social Support Appraisals scale [30]), and HIV care self-efficacy (modification to the HIV Treatment Adherence Self-Efficacy scale [31]). We also tested for significant differences in baseline clinical outcomes, including CD4 count, HIV viral suppression, and engagement in care. All analyses were conducted using SAS version 9.4.

#### Change in clinical outcomes

We plotted the proportion of members who were virally suppressed and engaged in care from baseline to each 6-month follow-up time point and assessed these changes using McNemar’s tests. We then classified all members as high or low PL users based on app use in the prior 6-months, with high users defined as members who had a 48% or greater response rate to daily queries in the past six months. A user’s PL use category could vary at each six month interval. We assessed for differences in viral suppression and engagement in care among high vs. low PL users at each 6-month interval using chi-square tests.

As a sensitivity analysis, we changed the usage cutoff from a 48% response rate to 90%, 75%, and 25% to determine whether differences in the clinical outcomes persisted with different definitions of high PL use.

#### Time to viral suppression

Among members with an unsuppressed HIV viral load at baseline (defined as the most recent lab value within 90 days prior to PL enrollment), we conducted a time-to-event analysis to estimate the average time to viral suppression from the date of PL enrollment. Members were censored upon reaching the end of PL enrollment (due to study completion, death, dropout, or the end of the data collection period) or at the start of a one-year window with no lab values. We estimated the association between PL use and time to achieve viral suppression using a Cox proportional hazards model. PL use was dichotomized each month as high or low using a 48% response rate in the previous month to identify high users. Members were eligible for inclusion in the analysis if they had at least one lab value after PL enrollment. Members were excluded if they achieved viral suppression in the first month following enrollment, because they did not have sufficient PL use to be classified as exposed. We adjusted for baseline covariates that improved model fit, as determined by lowest Akaike information criteria (AIC). Baseline covariates considered for the model included age, sex, race, insurance, education, income, perceived stress, social support, stigma, and confidence to adhere to medication.

As a sensitivity analysis, we examined time-to-viral suppression only among patients who had been diagnosed with HIV for at least six months to account for increases in viral suppression that might be observed for newly diagnosed patients who are first initiating care.

#### Sustained viral suppression

We examined sustained viral suppression by calculating the proportion of total follow-up time that members had a viral load <200 copies/mL in a 24-month time period. Members were included in the analysis if they had at least two viral load measurements in the 24 months following PL enrollment and had no more than a 12-month gap without a measurement. We fit a negative binomial regression model to estimate the association between PL use and sustained viral suppression, where PL use was treated as a continuous exposure based on cumulative response rates to queries at 24 months and sustained viral suppression was defined as the number of days with a suppressed viral load in the 24 month period. Virally suppressed days were counted as each day between two suppressed lab values, plus half the days between a suppressed and unsuppressed lab value. We adjusted the model for baseline age, sex, race, insurance, education, income, perceived stress, social support, stigma, and confidence to adhere to medication.

#### Engagement in care

Among members who were not meeting the HRSA engagement metric at study baseline, including those who were new to care and those who were not engaged in care, we examined the association between PL use and likelihood of becoming engaged in care. Members were included in the analysis if they had a least six months of PL use. Chart reviews facilitated the determination of HRSA metric achievement, therefore members were censored only upon reaching the end of PL enrollment (due to study completion, death, dropout, or the end of the data collection period). Both engagement in care and PL use were time-updated every six months. Because engagement in care was measured in intervals, we fit a discrete time proportional hazards model to estimate the association between PL use and engagement in care. As with the time-to-viral suppression analysis, covariates in the adjusted model were chosen among the full list of baseline characteristics based on lowest AIC.

## Results

Between September 2013 and March 2017, 127 members enrolled in PL. Nearly two thirds (65%) were male, about half (53%) were non-Hispanic Black, and the median age was 39 (interquartile range 29–50). Most members had health insurance, with 42% covered by public insurance and 30% covered by private insurance. Over half of members (52%) earned less than 50% of the federal poverty level, and 80% of members completed at least a high school education. Most members (63%) reported high levels of stress on the Perceived Stress Scale and moderate levels of HIV-related stigma (mean = 101 on a scale from 40 to 160). Members also reported high levels of confidence in their ability to start and maintain HIV care, with confidence levels ranging from 69% to 84% for various aspects of HIV care. At baseline, 75 members (60%) were virally suppressed and 76 (60%) met the HRSA metric for engagement in care. Baseline demographic and clinical characteristics did not differ significantly among members who had high vs. low PL use in the first month of enrollment (Table 1).

**Table 1:** Baseline participant characteristics.

	<48% PL Use at 1 Month N = 36	≥48% PL Use at 1 Month N = 91	p-value
**Age in years, mean (SD)**	39.8 (14.8)	39.0 (12.0)	0.76
**Gender, n (%)**			0.89
Male	25 (69.4)	58 (63.7)	
Female	10 (27.8)	30 (33.0)	
Transgender M-F	1 (2.8)	2 (2.2)	
Missing	0 (0)	1 (1.1)	
**Race/Ethnicity, n (%)**			0.96
White non-Hispanic	10 (27.8)	28 (30.8)	
Black non-Hispanic	19 (52.8)	48 (52.8)	
Hispanic	2 (5.6)	5 (5.5)	
Other	5 (13.9)	10 (11.0)	
**Insurance status, n (%)**			0.87
Public	16 (44.4)	37 (40.7)	
Private	9 (25.0)	29 (31.9)	
None	10 (27.8)	21 (23.1)	
Other	1 (2.8)	4 (4.4)	
**Income compared to FPL, n (%)**			0.60
<50% FPL	17 (47.2)	48 (52.8)	
50–100% FPL	9 (25.0)	15 (16.5)	
≥100% FPL	10 (27.8)	25 (27.5)	
Missing	0 (0)	3 (3.3)	
**Level of Education, n (%)**			0.30
Less than high school	9 (25.0)	17 (18.7)	
High school or equivalent	18 (50.0)	35 (38.5)	
More than high school	9 (25.0)	37 (40.7)	
Missing	0 (0)	2 (2.2)	
**Perceived stress, n (%)**			0.78
Low	2 (5.6)	3 (3.3)	
Moderate	10 (27.8)	30 (33.0)	
High	24 (66.7)	56 (61.5)	
Missing	0 (0)	2 (2.2)	
**HIV Stigma Scale**^**1**^**, mean (SD)**	105.0 (15.9)	99.7 (19.5)	0.15
Missing, n (%)	0 (0)	2 (2.7)	
**Social Support Appraisal Scale**^**2**^**, mean (SD)**	44.4 (14.4)	43.7 (11.4)	0.82
Missing, n (%)	11 (30.6)	41 (45.1)	
**HIV care self-efficacy**^**3**^**, mean (SD)**			
Starting HIV care self-efficacy	79.5 (25.0)	83.8 (16.4)	0.44
Staying in care self-efficacy	175.8 (31.1)	176.9 (24.7)	0.87
HIV appointment self-efficacy	143.2 (37.9)	146.7 (26.8)	0.68
HIV medication self-efficacy	155.8 (25.4)	155.3 (28.4)	0.93
Missing, n (%)	11 (30.6)	39 (42.9)	
**Recent HIV diagnosis**^**4**^**, n (%)**			0.61
Yes	5 (13.9)	17 (18.7)	
No	31 (86.1)	74 (81.3)	
**Months from HIV diagnosis to enrollment, mean (SD)**	94.5 (95.6)	87.0 (96.6)	0.70
**HIV viral suppression (<200), n (%)**			0.16
Yes	17 (47.2)	58 (63.7)	
No	18 (50.0)	31 (34.1)	
Missing	1 (2.8)	2 (3.7)	
**Engaged in care, n (%)**			0.84
Yes	21 (58.3)	55 (60.4)	
No	15 (41.7)	36 (39.6)	

Table 1 displays baseline characteristics of the study sample (N = 127) by PL usage. PL use was determined by participant’s average response rate to three daily queries (mood, stress, and medication adherence) in the first month of PL enrollment.

^1^Scores on the Berger HIV Stigma scale range from 40 (low stigma) to 160 (high stigma)

^2^Scores on the Social Support Appraisals Scale range from 23 to 92, with a lower score indicative of a greater level of appraised social support

^3^HIV care self-efficacy scores range from 0 to 100 for starting HIV care and from 0 to 210 for staying in care, appointment self-efficacy, and medication self-efficacy, with higher scores indicative of greater self-efficacy

^4^Recent HIV diagnosis defined as within 6 months of PL enrollment

### App usage

PL users enrolled in PL on a rolling basis from September 2013 through March 2017, leading to variable follow-up times for members. Of the 127 PL users, 23 users were followed for less than 6 months (2 members died and 21 reached the end of the study prior to completing 6 months of follow-up). Three members dropped out of PL between their six- and twelve-month follow-up, and an additional 28 members reached the end of the study period prior to completing 12 months of follow-up. 73 members had 12 full months of enrollment. One additional member dropped out before meeting the two-year follow up, while 11 members reached the end of the study period. In total, 61 members completed 24 months of follow-up. S1 Table shows the number of members enrolled in PL over time.

Response rates to daily queries were calculated in 6-month intervals for members who received consistent queries (defined as receiving queries for at least 5.5 months in the 6-month interval). Cohort response rate to queries was highest in the first 6 months of PL use at approximately 60%. This rate declined at 6–12 months to approximately 40% and then remained stable, with a 50% response rate from 12–18 months and 45% from 18–24 months. The number of community board posts was also highest in the first 6 months, with a mean of approximately 19 posts per member. The mean number of community posts between 6–12 months of enrollment was approximately 7, and members made an average of about 15 community posts between months 18–24. Table 2 provides details on the app usage at 6-month intervals following enrollment.

**Table 2:** App utilization over time.

	N	Meds	Mood	Stress	CMB
		Mean Response Rate (SD)	Mean Response Rate (SD)	Mean Response Rate (SD)	Mean Posts per Person (SD)
**Baseline**	127	--	--	--	--
**6 months**	98	61.3 (38.1)	60.0 (38.8)	59.9 (38.8)	19.3 (66.2)
**12 months**	65	40.2 (41.7)	39.5 (41.8)	39.6 (41.7)	7.2 (16.6)
**18 months**	45	52.8 (38.4)	52.7 (38.5)	53.0 (38.7)	8.2 (13.5)
**24 months**	38	44.9 (38.0)	44.2 (37.5)	44.2 (37.7)	14.9 (36.4)

Table 2 shows the cohort response rate percentage for daily queries (medication, mood, and stress) and number of community board posts. Rates represent the average in the six-month period and are not cumulative (i.e. 6-month rates represent data from baseline through six months; 12-month rates represent data from six months through twelve months). Rates are only calculated for members who are missing less than 2 weeks of app data during the 6-month interval.

The second version of PL offered a messaging feature. In the first six month interval, patients sent an average of slightly more than one message per month, with slightly more messages sent among patients with a suppressed viral load. Among 69 patients who were virally suppressed at six months, the median number of messages sent was 2 (interquartile range (IQR) 0–8), with a mean of 7.7 messages. The five patients who were not virally suppressed at six months sent fewer messages on average, with a mean of 6.2 and a median and interquartile range of 1 and 0–13, respectively.

### Clinical outcomes

**Fig 1:**
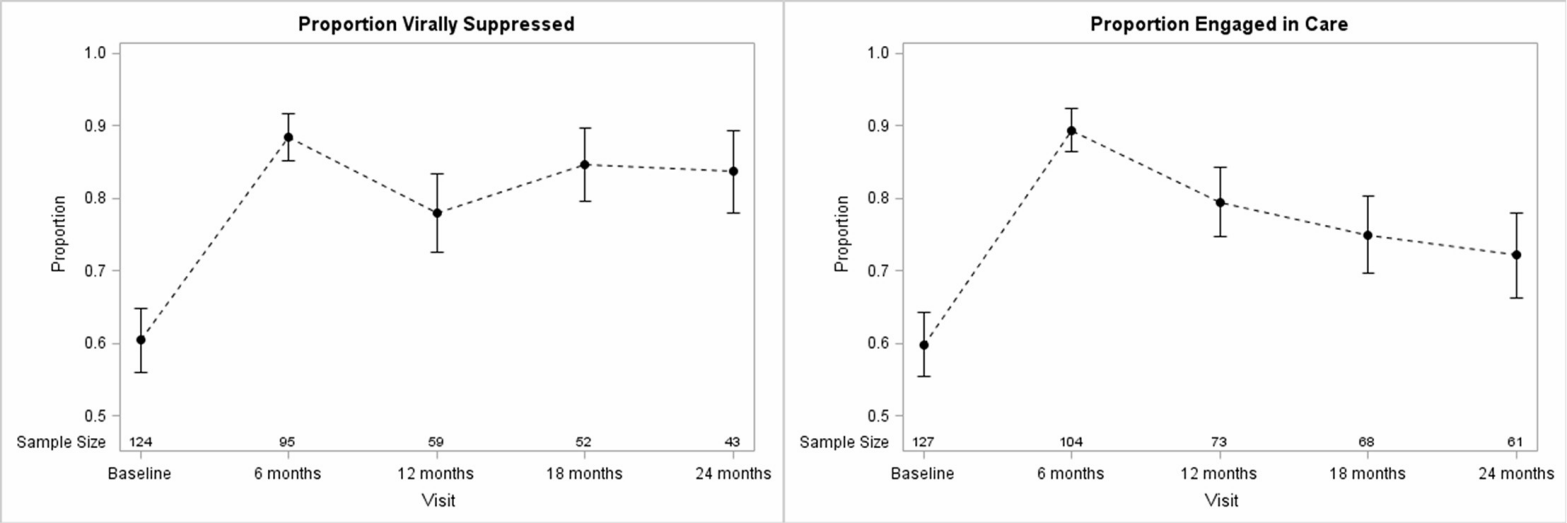
Change in clinical outcomes over time. Fig 1 shows the change in the viral suppression and engagement in care from baseline to each 6-month follow-up period. PL users are included in the sample for each time point during which they were still enrolled in PL. PL enrollment ended when members finished the study (N = 26), dropped out (N = 4), died (N = 2), or reached the end of the data collection period (N = 95). Viral suppression is defined as having a viral load <200 copies/mL. Engagement in care is defined as having attended 2 or more HIV appointments separated by at least 90 days within the past year. Increases in both viral suppression and engagement in care were statistically significant at each follow-up period compared to baseline (p<0.05).

Viral suppression and engagement in care both improved significantly from baseline to each 6-month follow-up interval. At the time of enrollment, 60% of PL members were virally suppressed and 60% met HRSA criteria for engagement in care. By six months, the proportion of virally suppressed members improved to 88% and the proportion engaged in care improved to 89%. The improvement persisted throughout the 24 month period. Fig 1 depicts the trends in both outcomes over time.

When further examining outcomes by levels of PL use, members who used PL with at least a 48% daily response rate showed better clinical outcomes at follow-up compared to those with lower PL use, despite having similar clinical markers at baseline. At six months, 95% of members with high PL use were virally suppressed compared to 79% of members with low PL use; this trend continued through 24 months of follow-up. Engagement in care was also notably higher among members with high PL use compared to low, with 93% of high PL users retained in care at 6 months compared to 87% of low users. This trend also persisted through 24 months of follow-up (Table 3).

**Table 3:** Clinical outcomes by PL use over time.

	Low PL Use		High PL Use		
**Viral Suppression**	**N**	**Percent Suppressed**	**N**	**Percent Suppressed**	**p-value**
**6 months**	33	78.8	53	94.6	0.03
**12 months**	30	76.7	23	87.0	0.48
**18 months**	15	86.7	22	90.9	0.99
**24 months**	13	69.2	18	94.4	0.13
**Engagement in Care**	**N**	**Percent Engaged**	**N**	**Percent Engaged**	**p-value**
**6 months**	39	87.2	59	93.2	0.48
**12 months**	38	73.7	27	88.9	0.21
**18 months**	22	77.3	23	95.7	0.10
**24 months**	20	75.0	18	88.9	0.41

Table 3 shows the observed outcomes by level of PL use. PL members are included in a given time point for which they were actively enrolled in PL, defined as receiving daily queries for at least 5.5 months of the 6-month interval. Members who responded to ≥48% of daily queries in the prior 6-months are classified as high PL users; those with <48% response rates are low users. Viral suppression is defined as having a viral load <200 copies/mL. Engagement in care is defined as having attended 2 or more HIV appointments separated by at least 90 days within the past year.

In a sensitivity analysis, we explored the impact of changing the query response rate used to classify high PL use. We tested for differences in viral suppression and engagement in care among people with high vs. low PL use considering response rates of 90%, 75%, and 25%. Changing the cutoff value to define high PL use did not change the patterns observed regarding the comparison between high vs. low PL users: high users continued to show better viral suppression and engagement in care. However, there may be a dose-response trend whereby increasing the cutoff for high use increases the proportion of members with viral suppression and engagement in care. This trend would require a larger sample size to determine significance. S2 Table presents the results of both sensitivity analyses.

#### Time to viral suppression

Among 49 members without viral suppression at baseline, 43 had at least one lab value within one year after PL enrollment. Of these, 6 members achieved viral suppression within the first month of enrollment and were dropped from the analysis, as the 1-month lag in PL use resulted in no PL exposure for these six members. The final sample size included 37 members. Of these, 30 (81%) achieved viral suppression following PL enrollment. In unadjusted trends, members with high PL use were approximately 70% more likely to achieve viral suppression compared to those with low PL use (HR = 1.69, 95% CI 0.77–3.71). After adjusting for race, education, and baseline perceived stress (covariates chosen using lowest AIC), members with high PL use were approximately twice as likely to achieve viral suppression compared to those with low PL use (HR = 2.09, 95% CI 0.64–6.88) (Table 4).

**Table 4:** Time to first viral suppression by PL use.

	Unadjusted Hazard Ratio (95% CI) N = 37	Adjusted Hazard Ratio^1^ (95% CI) N = 29
**PL Query Response Rate**		
Low	Ref	Ref
High	1.69 (0.77–3.71)	2.09 (0.64–6.88)

Among patients who were not virally suppressed at baseline, patients were followed from their date of PL enrollment until the first date on which they had a lab value indicating viral suppression (HIV viral load <200 copies/mL) or the date of censoring. Patients were censored for the following reasons: one year with no lab value, death, dropout, study completion, or administrative censoring on September 30, 2017. PL use is updated on a monthly basis and is calculated as the response rate to daily queries. A low query response rate is <48% and a high query response rate is ≥48%. PL use is lagged such that PL use in the month prior to the event of interest or censoring is treated as the exposure; patients who achieved viral suppression within the first month of PL enrollment were therefore considered unexposed and were not included in the analysis.

^1^Adjusted hazard ratio is adjusted for the following covariates: race (Black non-Hispanic, White non-Hispanic, Hispanic, Other/unknown), education (less than high school, high school or equivalent, greater than high school), and baseline perceived stress (low, moderate, high). Final adjusted model was chosen based on lowest AIC.

In a sensitivity analysis examining time-to-viral suppression among patients who had been diagnosed with HIV for at least six months, the unadjusted and adjusted hazard ratios were 1.68 (95% CI 0.65–4.35) and 2.06 (95% CI 0.31–13.81), respectively.

#### Sustained viral suppression

Sixty PL members had at least two lab values in the 24 months following PL enrollment and were included in the sustained viral suppression analysis. Of these, 29 (48.3%) were virally suppressed at baseline. Members maintained viral suppression for an average of 605.5 days in the two-year period (83% of total days). Greater PL use was associated with a greater likelihood of maintaining viral suppression in unadjusted analyses, with each 10% increase in response rate to daily queries associated with a 2.1% increase in number of days suppressed (incident rate ratio = 1.02, 95% CI 0.96–1.09). After adjustment, the incident rate ratio increased to 1.04 (95% CI 0.97–1.12) for each 10% increase in daily query response rate.

#### Engagement in care

Among 51 members who were not meeting the HRSA engagement metric at study baseline, 23 were new to care (i.e. diagnosed with HIV within one year of PL enrollment) and 28 had earlier diagnoses but were not retained in care. Twelve of these members had less than six months of PL use and were excluded from the analysis. Of the remaining 39 members, 35 (89.7%) achieved the HRSA metric during follow-up. Members with high PL use (>48% response rate) were more likely to achieve the HRSA metric than those with low PL use: 22 of 23 (95.7%) of high PL users achieved the HRSA metric, all within the first six months of enrollment, while 13 of 16 (81.3%) of low PL users achieved the HRSA metric, 10 within the first six months and 3 within the first year. The estimated association between PL use and engagement in care, after adjusting for gender, education, and income, was 1.52 (95% CI 0.89–2.57).

## Discussion

PLWH were more engaged in care and had better rates of viral suppression after enrolling in PL compared to baseline, with a statistically significant improvement through 24 months in both outcomes at each 6-month follow-up period. Though the results observed were exploratory and no causal conclusions can be drawn, we further observed that greater engagement with PL was associated with greater clinical benefit: participants with higher PL use were more likely to achieve and/or maintain viral suppression and to be engaged with care compared to members with lower PL use. These findings bolster the results from the PL pilot study, indicating that PL may be a useful long-term self-management tool for patients. This contributes to a strong preliminary evidence base and points to the need for a randomized trial to evaluate the causal impact of PL on long-term clinical outcomes such as sustained viral suppression.

Over 24 months of the study, usage of the app remained robust. PL use was high among most users, with a mean daily response rate of about 60% at six months, declining to about 45% at 2 years. Several members were very high users, with approximately one third of PL users maintaining an average query response rate over 90% in the first six months of use. A >90% response rate remained among approximately one in four members through 18 months and 15% of members at two years. These findings of sustained engagement through 24 months with an mHealth intervention have not previously been demonstrated. Most published mHealth interventions report 3 months follow-up, with a maximum of 12 months [32,33].

In this analysis we identified high users based on responses to daily queries, which represents the most direct indicator of daily PL use. However, PL contains many additional features beyond the daily queries with which participants may choose to engage. For example, some participants interact with the community board either by posting or reading others’ posts. Additionally, PL 2.0 introduced a messaging feature that allowed members to communicate securely with their provider. Finally, there are informational resources available within the app. This study did not capture any of that activity within the app and may therefore under-estimate app “usage.”

A limitation of this study is that all participants were PL users and, as such, we were unable to make individual-level comparisons to non-PL users. However, compared to clinic averages over the same time period, improvements in viral suppression rates and engagement in care were notably larger among PL users: clinic-level viral suppression increased only slightly from 87% to 90% and engagement in care declined among the full clinic from 85% to 81%. Another limitation is that in the absence of random assignment to PL use, it is possible that members with high response rates to daily queries were inherently more engaged in their health, resulting in both higher PL use and also better engagement in care and reduced viral load. Although we considered baseline self-efficacy measures as adjustment covariates in the analyses, there were no significant differences at baseline between high and low PL users on measures of HIV care self-efficacy and the addition of these variables did not significantly contribute to the models. Similarly, some improvement engagement in care may have been a result of participating in a research study; this impact could be better quantified in a randomized control trial with a placebo arm. Finally, as a clinic-deployed intervention, PL’s affiliation with the clinic may limit its generalizability to different care settings.

These results support and extend the findings of an earlier pilot study to 24 months of follow-up. They show improved engagement in care and viral suppression among PL users, with a trend toward better outcomes among members with greater engagement in PL indicating that more PL use may be associated with progressively better outcomes. The additional evidence reported here suggests a long-term benefit of PL on key clinical outcomes for PLWH including sustained viral suppression, an essential individual and population level indicator that improves the health of PLWH and contributes to the prevention of HIV transmission, both key factors for ending the epidemic. A larger, randomized clinical trial of PL will be important to determining the causal impact of PL more definitively.

## Supporting information

S1 Table PL enrollment over time. “Cumulative enrollments” describes the number of patients who had ever enrolled in PL by a given time point. PL members were “active users” beginning on their date of enrollment until one of the following: voluntarily unenrolled (N = 4), completed the study^1^ (N = 26), death (N = 2), or the data collection period ended (N = 95).
(DOCX)

S2 Table Clinical outcomes by PL use over time. PL members are included in all time points for which they were actively enrolled, defined as receiving daily queries for at least 5.5 months of the 6-month interval. Members who responded to ≥90% (Panel A), ≥75% (Panel B), or ≥25% (Panel C) of daily queries in the prior 6-months are classified as high PL users. Viral suppression is defined as having a viral load <200 copies/mL. Engagement in care is defined as having attended 2 or more HIV appointments separated by at least 90 days within the past year.
(DOCX)
