# Supplementary material for: Long term impact of PositiveLinks: Clinic-deployed mobile technology to improve engagement with HIV care
Source: PLoS One. 2020 Jan 6;15(1):e0226870. doi: 10.1371/journal.pone.0226870 (PMC6944340; doi:10.1371/journal.pone.0226870)
Supplement: S1 Table — “Cumulative enrollments” describes the number of patients who had ever enrolled in PL by a given time point. PL members were “active users” beginning on their date of enrollment until one of the following: voluntarily unenrolled (N = 4), completed the study1 (N = 26), death (N = 2), or the data collection period ended (N = 95). (DOCX) [file pone.0226870.s001.docx]

**S1 Table: PL Enrollment over Time**

“Cumulative enrollments” describes the number of patients who had ever enrolled in PL by a given time point. PL members were “active users” beginning on their date of enrollment until one of the following: voluntarily unenrolled (N=4), completed the study^1^ (N=26), death (N=2), or the data collection period ended (N=95).

|  | **Cumulative Enrollments** | **Total Active Users** | **Cumulative Inactive Users** | | | |
| --- | --- | --- | --- | --- | --- | --- |
|  |  |  | **Voluntarily Unenrolled** | **Completed Study** | **Death** | **Study End** |
| **2013-September** | 3 | 3 | 0 | 0 | 0 | 0 |
| **2013-December** | 26 | 26 | 0 | 0 | 0 | 0 |
| **2014-March** | 33 | 33 | 0 | 0 | 0 | 0 |
| **2014-June** | 45 | 43 | 2 | 0 | 0 | 0 |
| **2014-September** | 54 | 52 | 2 | 0 | 0 | 0 |
| **2014-December** | 62 | 59 | 2 | 0 | 1 | 0 |
| **2015-March** | 68 | 65 | 2 | 0 | 1 | 0 |
| **2015-June** | 77 | 73 | 3 | 0 | 1 | 0 |
| **2015-September** | 77 | 71 | 3 | 2 | 1 | 0 |
| **2015-December** | 77 | 58 | 3 | 15 | 1 | 0 |
| **2016-March** | 77 | 52 | 3 | 21 | 1 | 0 |
| **2016-June^2^** | 80 | 51 | 4 | 24 | 1 | 0 |
| **2016-September** | 103 | 62 | 4 | 26 | 2 | 9 |
| **2016-December** | 115 | 74 | 4 | 26 | 2 | 9 |
| **2017-March** | 127 | 86 | 4 | 26 | 2 | 95 |

*^1^Patients in PL 1.0 were enrolled for up to 24 months or until the launch of PL 2.0 in June 2016. Patients in PL 2.0 were enrolled for up to 12 months or until the end of the study on March 31, 2017.*

*^2^Note: PL 2.0 was introduced in June 2016*
