# Supplementary material for: Long term impact of PositiveLinks: Clinic-deployed mobile technology to improve engagement with HIV care
Source: PLoS One. 2020 Jan 6;15(1):e0226870. doi: 10.1371/journal.pone.0226870 (PMC6944340; doi:10.1371/journal.pone.0226870)
Supplement: S2 Table — PL members are included in all time points for which they were actively enrolled, defined as receiving daily queries for at least 5.5 months of the 6-month interval. Members who responded to ≥90% (Panel A), ≥75% (Panel B), or ≥25% (Panel C) of daily queries in the prior 6-months are classified as high PL users. Viral suppression is defined as having a viral load <200 copies/mL. Engagement in care is defined as having attended 2 or more HIV appointments separated by at least 90 days within the past year. (DOCX) [file pone.0226870.s002.docx]

**S2 Table: Clinical Outcomes by PL Use over Time**

PL members are included for all time points in which they were actively enrolled, defined as receiving daily queries for at least 5.5 months of the 6-month interval. Members who responded to ≥90% (Panel A), ≥75% (Panel B), or ≥25% (Panel C) of daily queries in the prior 6-months are classified as high PL users. Viral suppression is defined as having a viral load <200 copies/mL. Engagement in care is defined as having attended 2 or more HIV appointments separated by at least 90 days within the past year.

**Panel A: 90% Cutoff**

|  | Low PL Use: <90% | | High PL Use: ≥90% | |  |
| --- | --- | --- | --- | --- | --- |
| Viral Suppression | **N** | **Percent Suppressed** | **N** | **Percent Suppressed** | **p-value** |
| 6 months | 53 | 83.0 | 36 | 97.2 | 0.04 |
| 12 months | 37 | 75.7 | 16 | 93.8 | 0.25 |
| 18 months | 25 | 88.0 | 12 | 91.7 | 0.99 |
| 24 months | 25 | 80.0 | 6 | 100.0 | 0.55 |
| Engagement in Care | **N** | **Percent Engaged** | **N** | **Percent Engaged** | **p-value** |
| 6 months | 61 | 86.9 | 37 | 97.3 | 0.15 |
| 12 months | 47 | 74.5 | 18 | 94.4 | 0.09 |
| 18 months | 33 | 81.8 | 12 | 100.0 | 0.17 |
| 24 months | 32 | 81.3 | 6 | 83.3 | 0.99 |

**Panel B: 75% Cutoff**

|  | Low PL Use: <75% | | High PL Use: ≥75% | |  |
| --- | --- | --- | --- | --- | --- |
| Viral Suppression | **N** | **Percent Suppressed** | **N** | **Percent Suppressed** | **p-value** |
| 6 months | 42 | 83.3 | 47 | 93.6 | 0.18 |
| 12 months | 37 | 75.7 | 16 | 93.8 | 0.25 |
| 18 months | 17 | 88.2 | 20 | 90.0 | 0.99 |
| 24 months | 20 | 75.0 | 11 | 100.0 | 0.13 |
| Engagement in Care | **N** | **Percent Engaged** | **N** | **Percent Engaged** | **p-value** |
| 6 months | 49 | 87.8 | 49 | 93.9 | 0.49 |
| 12 months | 47 | 74.5 | 18 | 94.4 | 0.09 |
| 18 months | 24 | 79.2 | 21 | 95.2 | 0.19 |
| 24 months | 27 | 81.5 | 11 | 81.8 | 0.99 |

**Panel C: 25% Cutoff**

|  | Low PL Use: <25% | | High PL Use: ≥25% | |  |
| --- | --- | --- | --- | --- | --- |
| Viral Suppression | **N** | **Percent Suppressed** | **N** | **Percent Suppressed** | **p-value** |
| 6 months | 24 | 83.3 | 65 | 90.8 | 0.45 |
| 12 months | 25 | 80.0 | 28 | 82.4 | 0.99 |
| 18 months | 10 | 80.0 | 27 | 92.6 | 0.29 |
| 24 months | 10 | 70.0 | 21 | 90.5 | 0.30 |
| Engagement in Care | **N** | **Percent Engaged** | **N** | **Percent Engaged** | **p-value** |
| 6 months | 28 | 89.3 | 70 | 91.4 | 0.71 |
| 12 months | 33 | 69.7 | 32 | 90.6 | 0.06 |
| 18 months | 14 | 78.6 | 31 | 90.3 | 0.36 |
| 24 months | 16 | 75.0 | 22 | 86.4 | 0.43 |
